# Novel Strategies for Spinal Cord Regeneration

**DOI:** 10.3390/ijms23094552

**Published:** 2022-04-20

**Authors:** Bogdan Costăchescu, Adelina-Gabriela Niculescu, Marius Gabriel Dabija, Raluca Ioana Teleanu, Alexandru Mihai Grumezescu, Lucian Eva

**Affiliations:** 1 “Gr. T. Popa” University of Medicine and Pharmacy, 700115 Iasi, Romania; costachescus@gmail.com (B.C.); mariusdabija.md@gmail.com (M.G.D.); 2 “Prof. Dr. N. Oblu” Emergency Clinical Hospital, 700309 Iasi, Romania; elucian73@yahoo.com; 3 Department of Science and Engineering of Oxide Materials and Nanomaterials, Faculty of Applied Chemistry and Materials Science, Politehnica University of Bucharest, 011061 Bucharest, Romania; adelina.niculescu@upb.ro; 4 Department of Pediatric Neurology, “Dr. Victor Gomoiu” Children’s Hospital, 022102 Bucharest, Romania; raluca.teleanu@umfcd.ro; 5 “Carol Davila” University of Medicine and Pharmacy, 020021 Bucharest, Romania; 6 Research Institute of the University of Bucharest—ICUB, University of Bucharest, 050657 Bucharest, Romania; 7 Academy of Romanian Scientists, Ilfov No. 3, 050044 Bucharest, Romania

**Keywords:** spinal cord injury, delivery nanosystems, biomaterial scaffolds, stem cells, tissue engineering, spinal cord regeneration

## Abstract

A spinal cord injury (SCI) is one of the most devastating lesions, as it can damage the continuity and conductivity of the central nervous system, resulting in complex pathophysiology. Encouraged by the advances in nanotechnology, stem cell biology, and materials science, researchers have proposed various interdisciplinary approaches for spinal cord regeneration. In this respect, the present review aims to explore the most recent developments in SCI treatment and spinal cord repair. Specifically, it briefly describes the characteristics of SCIs, followed by an extensive discussion on newly developed nanocarriers (e.g., metal-based, polymer-based, liposomes) for spinal cord delivery, relevant biomolecules (e.g., growth factors, exosomes) for SCI treatment, innovative cell therapies, and novel natural and synthetic biomaterial scaffolds for spinal cord regeneration.

## 1. Introduction

The spinal cord represents the main communicating system between the brain and the body, ensuring information and signal exchanges for coordinating activities [1,2]. Injuring the spinal cord results in disrupting neural circuitry and connectivity, producing neurological disability [3].

Therefore, spinal cord injury (SCI) is one of the most complicated diseases, due to its pathological consequences affecting sensory, motor, and/or autonomic functions [4,5,6]. Specifically, SCIs could cause impairment in blood flow, breathing, temperature, body pressure, and sensory appendages, leading to permanent consequences, such as paralysis, autonomic dysfunctions, and neuropathic pain [1,7].

Moreover, SCI is a frequent condition, affecting more than 20 million people worldwide, increasing ~700,000 patients per annum [8]. The treatment and rehabilitation processes required after SCI are costly and exhausting, posing a huge burden on patients, families, and healthcare systems. Treatment must be started as soon as possible after the trauma occurs, with acute care and early surgical interventions. However, these procedures are usually not enough to completely restore spinal cord functions. In such cases, additional treatment is needed for dealing with chronic sensory, motor, and reflex dysfunctions, which can take years of procedures and specific exercises, and even life-long treatment in the home environment [9,10,11].

In this context, it is important to research better performing therapeutic alternatives geared towards reducing treatment duration and improving its results. Thus, increasing research interest has been noticed in designing advanced repair strategies for spinal cord regeneration. Particular attention has been drawn to biomaterials and nanotechnology-enabled products for the controlled delivery and sustained release of various moieties, including drugs, bioactive molecules, imaging agents, and cells [12,13,14,15,16,17,18].

Aiming to set a framework for future research, this paper briefly describes the characteristics of SCI, further reviewing the newest developments reported in spinal regeneration, counting innovative nanocarrier systems, biomolecules of interest for SCI treatment, recent cell therapies, and promising cell-free and cell-seeded biomaterial scaffolds.

## 2. Spinal Cord Injury

**Figure 1:**
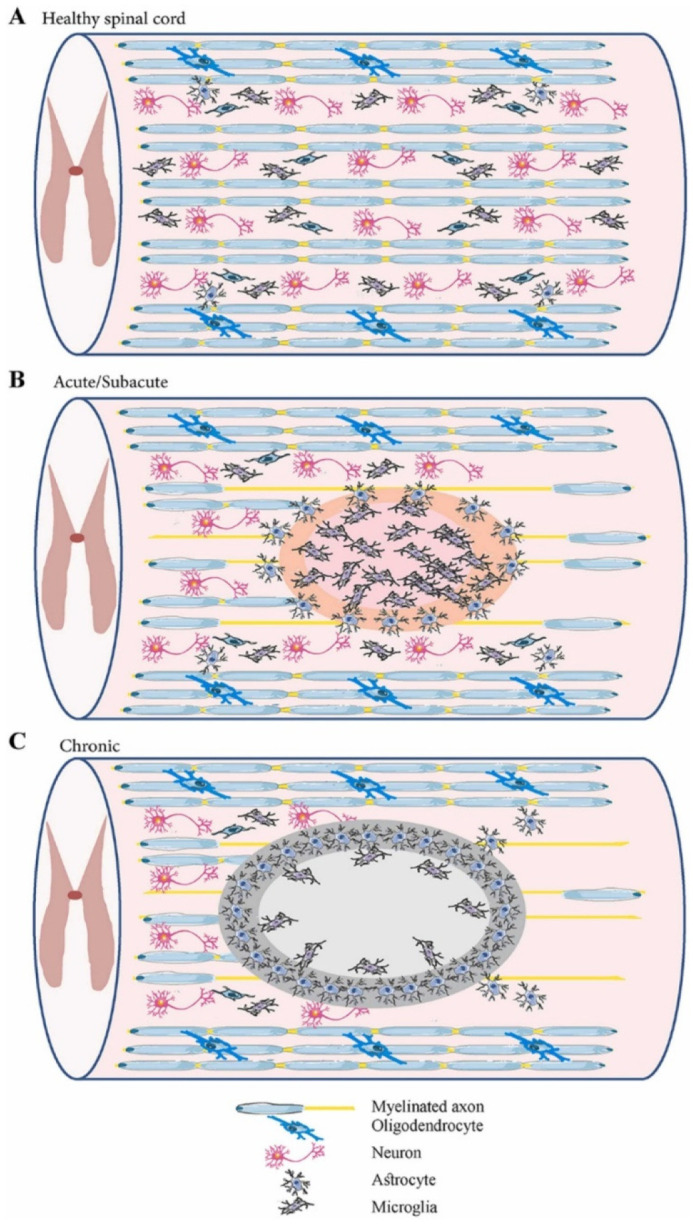
Schematic representation of the spinal cord in different phases: (**A**) healthy spinal cord; (**B**) acute/subacute injury phase (marked by cell necrosis originating from the spinal cord injury (SCI) and activation of inflammatory processes); (**C**) chronic phase (marked by cyst cavity formation and lesion expansion). Reprinted from an open-access source [21].

Spinal cord injury (SCI) is one of the most devastating traumas, as it can interrupt the connection between the brain and peripheral organs, producing complex pathophysiology [2,8,11]. SCI can be triggered by a broad range of physical impacts, including traffic accidents, falls, sports injuries, industrial accidents, and violent acts in which bony and ligamentous structures are injured and lose their protective ability. Thus, impact load is transferred through neurological structures [8,19,20]. The external insult is reflected in primary spinal tissue damage and neural cell death in the acute phase, while a subsequential secondary cascade of degenerative events is started [4,18,20,21,22,23] (Figure 1). The main characteristics of primary and secondary injuries are comprised in Table 1.

**Figure 2:**
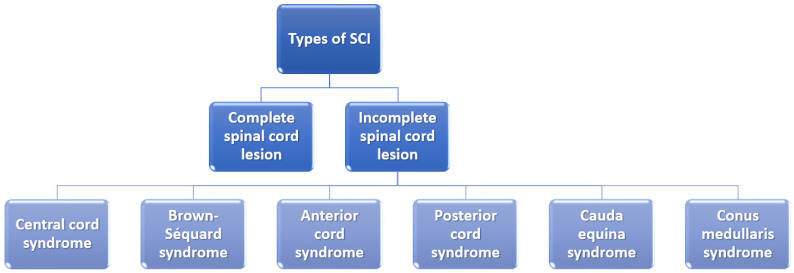
Classification of spinal cord injuries. Created based on information from [6,25].

It is also essential to mention that the severity of the SCI depends on whether the lesion is complete or incomplete and, if it is incomplete, what part of the spinal cord is affected (Figure 2). In the case of a complete spinal cord lesion, there is no preservation of any motor and/or sensory function at more than three segments below injury level in the absence of spinal shock. On the other hand, incomplete spinal cord lesion patients preserve residual motor or sensory function to some extent at more than three segments below the level of the injury [6,24].

However, intervention is required as soon after trauma as possible, irrespective of the type of SCI, in order to avoid secondary injury [19]. The main current treatment strategies include the administration of high-dose methylprednisolone sodium succinate, surgical interventions for anatomically stabilizing and decompressing the spinal cord, and rehabilitative care. Nonetheless, these therapeutic approaches have modest outcomes, imposing the need to develop novel treatment alternatives that would considerably improve patients’ quality of life [21,26].

## 3. Advanced Repair Strategies

**Figure 3:**
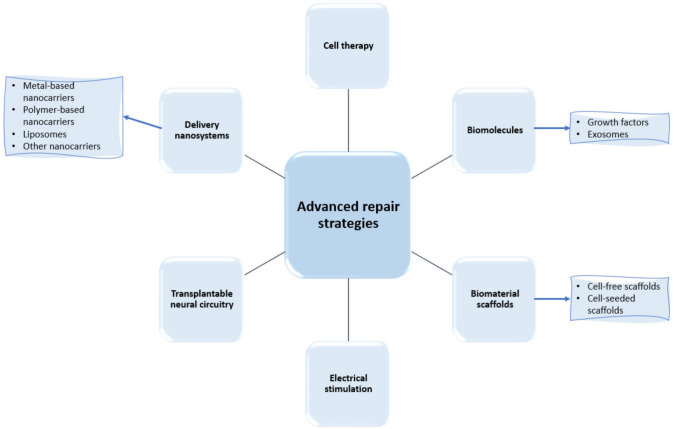
Examples of recently researched spinal cord regeneration strategies.

In their research efforts to find more performant treatment strategies for spinal cord regeneration, scientists have recently focused their works on a series of advanced repair strategies, as presented in Figure 3. Thus, the following subsections comprehensively describe each category of these approaches, with a particular focus on the developments of delivery nanosystems and biomaterial scaffolds.

### 3.1. Delivery Nanosystems

Most therapeutic agents implied in treating traumatic central nervous system injuries face limitations in reaching the target site and exerting their intended pharmacological effects. The obstacles encountered by drugs include physiological barriers (e.g., blood–brain barrier, blood–spinal cord barrier), instability in physiological conditions, rapid elimination from the injured tissue or cerebrospinal fluid, and off-target toxicity [27,28,29].

One of the biggest challenges in treating SCIs remains the blood–spinal cord barrier. This physical barrier between the blood and spinal cord parenchyma prevents toxins, blood cells, and pathogens from entering, and the spinal cord becomes an obstacle for therapeutic agents’ delivery to the injured site [30,31]. This issue can be solved by opening this barrier through various modalities (e.g., magnetic resonance imaging-guided focused ultrasound [32], miRNA-125a-5p silencing [33]) or by creating smart medicines able to penetrate it.

Thus, increasing focus has been directed to creating central nervous system (CNS) delivery systems that would allow a more efficient treatment. Particularly, nanomaterials of various compositions have been reported as promising nanocarriers, overcoming low drug permeability and ensuring targeted and controlled release. A large variety of nanoparticulate systems can be loaded with drugs, imaging moieties, enzymes, cell components, and other biomolecules. The ingenious design of nanovehicles brings unprecedented advantages in terms of reducing cargo degradation, improving drug absorption, promoting selective interactions with cell compartments, diminishing adverse effects, and increasing the bioavailability of drugs [1,34,35,36]. Hence, delivery nanosystems represent an attractive strategy for SCI treatment, as they can improve recovery time by targeting localization, altering the signaling pathways and cellular uptake [1].

In this respect, numerous nanomaterials have been investigated, leading to encouraging results. The following subsections discuss some of the most recent strategies for SCI management, classified according to nanocarrier type.

#### 3.1.1. Metal-Based Nanocarriers

Metal-based nanomaterials represent an attractive possibility for encapsulating and delivering bioactive molecules to the site of interest [37,38]. Particularized for reaching the place of an SCI lesion, gold has been noticed as the most investigated nanoscale metal. Gold-based nanocarriers are advantageous especially due to their stability, biocompatibility, low cytotoxicity, ability to regulate cell growth pathways, and the possibility of surface functionalization [39,40,41].

For instance, Fang et al. [42] have biosynthesized gold nanoparticles (Au NPs) from the bark extract of *Juglans regia* and further loaded them with zonisamide. The particles offered high stability and solubility under room temperature conditions, ensuring a controlled release of the incorporated drug. The authors concluded that the as-designed nanomedicine could serve as a promising clinical drug for future spinal cord injury repair applications.

Alternatively, Zhou et al. [34] reported the conjugation of gold nanoclusters with herbal medicines (i.e., berberine, astragalus polysaccharides, and diosgenin) towards generating enhanced neuroprotective and anti-neuroinflammation effects. The nanosystems inhibited inflammation factors, such as NF-κB and IKKβ, reducing inflammatory responses and recovering nerve functions after SCI.

Pursuing a similar aim, Kim et al. [43] have fabricated Au NPs conjugated with β-cyclodextrin loaded with ursodeoxycholic acid. The as-designed complex was noted to considerably decrease pro-inflammatory cytokines and increase anti-inflammatory cytokines compared to free anti-neuroinflammatory drugs. Moreover, the nanosystem suppressed the phosphorylation of ERK and JNK in the MAPK pathway, expressed inducible nitric oxide synthase, and induced the expression of arginase-1, consequently being considered a promising alternative drug system for SCI cases.

A different approach has been recently proposed by Lin et al. [36]. The authors reported the preparation of red fluorophore gold nanodots caped with glutathione that can be employed as computed tomography (CT) contrast agents for better visualizing the spinal cord. The nanosystem exhibited remarkable photoluminescence stability and a high attenuation coefficient to X-rays, being a useful tool for avoiding the high toxicity and weak CT signal of traditional iodine contrast.

In addition to gold, other metal-based nanoproducts can be of good use in this biomedical field [44]. For example, Wang and colleagues [45] exploited the potential of polyethylene glycol (PEG)-modified maghemite nanospheres. The authors loaded the nanoparticles with tacrolimus, assuming this drug’s possible action on the signaling pathway through macrophage polarization M1 and M2 and induction of neuronal cell growth. The nanosystem was noted to augment the tacrolimus effect, enhance the locomotor activity of model animals, accelerate their recovery time, reduce oxidative stress, IL-6, IL-2, and TNF-α inflammatory factors, and the reduce the expression ratio of M1 to M2.

To summarize the characteristics of the above-discussed nanosystems in a clear and concise manner, Table 2 was created.

#### 3.1.2. Polymer-Based Nanocarriers

Attracted by the variety and versatility of polymers, many researchers opted to deliver therapeutic agents via polymer-based nanocarriers. These materials benefit from good solubility, stability, safety, and controlled cargo release with slow degradation [46,47,48].

Poly(lactic-co-glycolic acid) (PLGA) nanoparticles started to gather attention for treating traumatic CNS injuries [21]. For example, Azizi et al. [49] prepared PLGA nanospheres loaded with the ChABC enzyme for SCI therapy. Given that this nanoparticulate treatment led to myelin formation and glial scar degradation in model animals, these nanosystems represent a suitable candidate for spinal cord repair, functional recovery, and axonal regeneration. Similarly, Andrabi et al. [23] utilized PLGA-based biodegradable nanoparticles for the delivery of antioxidant enzymes (i.e., superoxide dismutase, catalase). These nanosystems could protect lesion site mitochondria from oxidative stress and subsequential secondary injury. Moreover, the as-designed nanoparticles prevented the release of cytochrome c, inhibited activation of Caspase-3, and protected the spinal cord from cell apoptosis and further degeneration. Thus, it can be a useful nanomedicine in the early stage of SCI, minimizing the impact of primary injury response and improving neurological and functional recovery over time.

Interesting results have also been reported for other polymer-based nanosystems. Liu et al. [50] have prepared electrospun fibrous mats made of polylactic acid (PLA) fibers encapsulated with docosahexaenoic acid (DHA). The polymeric nanomats presented adequate mechanical properties for sustained release of the drug, proving effective for SCI repair. More specifically, the nanosystem promoted neurite outgrowth in vitro and up-regulated neural marker genes BDNF and NT-3, leading to pronounced neurological function recovery. Thus, the as-designed nanocarrier can provide both the mechanical and chemical support required to fabricate future central nerve grafts.

Differently, Nie et al. [51] have fabricated nanomicelles from a tri-block copolymer (i.e., poly (ethylene glycol)-block-poly (propylene glycol)-block-poly (ethylene glycol) (PEG/PPG/PEG)). The scientists loaded PEG/PPG/PEG nanomicelles with zonisamide, creating a delivery system with controlled release at physiological pH. The nanoconstructs demonstrated enhanced antioxidant activity in spinal cord neurons stimulated by H_2_O_2_, holding great promise as candidates for SCI functional integrated therapy.

Natural polymers were also successfully employed in designing nanovehicles for spinal cord delivery. For instance, Wang et al. [52] have developed sesamol-loaded stearic acid-chitosan nanomicelles that were noted to ensure sustained drug release at physiological pH, potential dissolution rate, and stability for up to 15 days. In addition, these nanostructures exhibited better results than the free drug in regulating cell survival, membrane leakage, reactive oxygen species (ROS) generation, the activity of antioxidant systems, and apoptotic and inflammatory signaling pathway. These outcomes encouraged the authors to recommend the designed nanoplatforms to mitigate oxidative-stress-mediated apoptosis in neural cells. Alternatively, Fang and Song [53] used chitosan as a nanoshell to deliver CeO_2_ nanoparticles. The nanosystem was observed to increase auto-regenerative and neuroprotective activity in spinal cord regeneration, being a promising biocompatible material for SCI repair.

For clarity, Table 3 summarizes the characteristics of the above-presented polymer-based nanosystems.

#### 3.1.3. Liposomes

Liposomes have also attracted attention for creating effective delivery nanosystems. These spherical vesicles can protect the payload from degradation, ensuring its accumulation at the lesion site and enhancing therapeutic effects. Moreover, the ease of functionalization of these structures renders liposomes suitable for creating biomimicking nanoconstructs [54,55].

One example of liposomal formulation for spinal cord regeneration is offered by Zhang et al. [56]. The authors fabricated vitamin E succinate-grafted ε-polylysine nanoparticles pre-compressed with pOXR1 and loaded into cationic liposomes. These nanoconstructs protected DNA against DNase I degradation, maintaining its activity and successfully transporting the cargo into cells. Thus, the nanosystem reduced neural apoptosis, attenuated oxidative stress, and inhibited inflammation, promoting functional recovery in acute traumatic SCI.

A different approach is proposed by Tang et al. [57], who have fabricated macrophage membrane-camouflaged liposomes encapsulated with minocycline. The nanocarrier was noted to prolong drug circulation time, accumulate at the trauma site of the spinal cord, enhance the therapeutic effect, and exhibit anti-pyroptosis activity. Hence, this biomimetic strategy paves the way for new SCI targeting and treatment avenues.

Alternatively, Wang and colleagues [58] have focused their research on the relation between curative therapies for SCIs and intestinal complications. In this respect, the authors developed a gut–CNS axis-targeted delivery system consisting of neuropeptide apamin, stabilized by sulfur replacement with selenium, incorporated in a liposome covered by a non-covalent cross-linked chitosan oligosaccharide lactate layer. The nanoconstruct was reported to permeate through oral absorption barriers and, after systemic circulation, target local enteric glial cells and astrocytes. Thus, these nanoplatforms hold great promise for comprehensive SCI therapy.

Table 4 concisely presents the above-discussed liposome-based nanosystems from the point of view of their characteristics.

#### 3.1.4. Other Nanocarriers

Several other nanomaterials were reported in the literature as potential candidates for innovative and performant spinal cord therapy. For instance, nanogels attracted scientific interest in the field due to their capacity to reach the smallest capillary vessels, penetrate tissues via transcellular pathways, and release drug freight at the injury site [59]. Taking into account these advantageous properties, Papa et al. [60] designed a functionalized nanogel-based nanovector loaded with Rolipram that demonstrated selectivity towards astrocytes and limited macrophage uptake. After internalizing into the astrocytes’ cytoplasm, the nanogels (made of polyethylene glycol and linear polyethyleneimine) underwent lysosomal degradation, releasing the therapeutic anti-inflammatory freight. The authors concluded that other molecules or compounds could be incorporated as well, opening the door for advanced therapy of the inflammatory response in SCIs or other neurodegenerative diseases.

On a different note, Yu et al. [22] have recently reported the fabrication of a metformin-containing gelatin nanogel encapsulated into glutathione-modified macrophage-derived cell membranes. This biomimetic approach was tackled as a method of crossing the blood–spinal cord barrier and successfully reaching the injury site. The nanosystem showed a slow-release effect, good accumulation at the target site, and promising therapeutic effect in attenuating oxidative stress, inflammation, and apoptosis.

An interesting approach is offered by Mahya et al. [61], who have encapsulated berberine into chitosan nanoparticles and further included them in an alginate–chitosan hybrid hydrogel. The composite nanomaterial encountered proper swelling, degradability, and bioactivity, being a suitable microenvironment for spinal cord tissue engineering and controlled drug released systems.

Another recent study conducted by Wu and colleagues [62] presented a highly intelligent nanocarrier system for SCI repair. The authors created a stretched inverse opal film (SIOF) infiltrated with a temperature-sensitive hydrogel loaded with black phosphorus quantum dots (BPQDs), fibroblast growth factor 10 (FGF10), and chloroquine phosphate. This complex nanosystem exhibited the synergistic outcomes from the excellent photothermal effect of BPQDs: controlled cargo release, enhanced biocompatibility, and unique topography of the SIOF that could orientate cell growth and promote cytoskeleton elongation. Moreover, the nanoplatform exhibited excellent inflammation inhibition and promoted axon growth, aiding in the recovery of motor function in rat models.

Encouraging results have also been obtained by using drug-loaded nanovesicles. Particularly, Liu et al. [63] have recently prepared a nanovesicle derived from a macrophage membrane encapsulated with sodium alginate and naloxone. The nanosystem successfully accumulated at the lesion site, enhancing drug concentration in the traumatic area, thereby decreasing free Ca^2+^ concentration, alleviating the inflammatory response, and attenuating neural apoptosis. Moreover, the motor function of the treated mice significantly improved, demonstrating great potential for SCI treatment.

The above-described nanosystems are synthesized in Table 5.

### 3.2. Biomolecules

#### 3.2.1. Growth Factors

Growth factors have been increasingly studied in relation to spinal cord regeneration. They can regulate neurons’ survival, stimulate the release of neurotransmitters and recovery of synaptic function, promote the growth and remodeling of axons, and regenerate nerve cells. Particularly, neurotrophin-3 (NT-3), neurotrophin-4 (NT-4), and neurotrophin-5 (NT-5) have been recognized for their ability to protect damaged neurons and promote neuron growth and differentiation [18,21]. Interesting studies have been reported on the delivery of NT-3 to injured spinal cord regions [64]. For instance, Oudega et al. [65] have successfully implanted a chitosan-based implant for NT-3 release in the completely transected spinal cord of rats, observing the potential of this strategy for facilitating neural tissue generation. Differently, Cong and colleagues [66] concluded that direct intraspinal administration of NT-3 could inhibit excessive autophagy of oligodendrocytes after SCI, promoting the recovery of motor function.

Nerve growth factor (NGF) was also noticed to have an important role in spinal cord regeneration, supporting the neurons’ survival, growth, differentiation, and synapse formation, thereby stimulating nerve regeneration, remodeling the neural network, and aiding in the recovery of motor function [18,67,68]. Taking into account the beneficial actions of NGF, Yamanaka et al. [69] created a combination treatment for up-regulating this growth factor. Specifically, the authors used diazoxide and erythropoietin to attenuate spinal cord ischemic reperfusion and cytoarchitectural change via up-regulation of NGF expression. In addition, the researchers concluded that further studies of this mechanism could contribute to reducing spinal cord ischemic complications after the aortic intervention.

Alternatively, Cheng et al. [70] investigated the expression of NGF in activated astrocytes by a mouse model of contused spinal cord injury and in vitro studies. The authors noticed more astrocytes with immunoreactivity to proNGF in the injured spinal cord sites. Moreover, proNGF was reportedly localized in both exosome-like vesicles and the cytoplasm of astrocytes in culture, demonstrating that reactive astrocytes increased proNGF expression after SCI and indicating the potential association between exosome-like proNGF transport or release in inducing neuronal apoptosis and heightening SCI progression.

Valuable characteristics have also been reported for the brain-derived neurotrophic factor (BDNF). BDNF is recognized for its neuroprotective effects on 5-serotonin, dopaminergic, cholinergic, and GABA neurons via neuron growth promotion, axon sprouting and regeneration, and axon remyelination [18]. On the other hand, proBDNF may oppose the functions of mature BDNF through the inhibition of proliferation, differentiation, and migration of neural stem cells (NSCs) during development. Subsequentially, administrating anti-proBDNF antibody treatment was noted to promote NSC proliferation and differentiation [71].

#### 3.2.2. Exosomes

In recent years, the importance of intercellular communication has been emphasized through signaling organelles (i.e., extracellular vesicles). According to the size of these double-layered membrane vesicles, they can be divided into three classes: apoptotic bodies (1000–5000 nm), microvesicles (100–1000 nm), and exosomes (30–150 nm). Particular attention has been drawn to the small structures, as they can deliver information among cells in different pathological and physiological statuses [21,72].

Exosomes are important paracrine mediators of their parent cells that have been recently reported to hold great promise in medical treatments and tissue regeneration [73,74]. Exosomes are produced by the endosomal compartments of most cells, including those involved in immune and pro-inflammatory responses (e.g., macrophages, dendritic cells, and T and B lymphocytes). These vesicles can travel throughout the body towards target cells, where they participate in various biological and pathological processes, including tissue damage and repair responses [75]. Stem-cell-derived exosomes have been particularly investigated for their neuroprotective properties in several in vitro and in vivo studies in various therapeutic approaches [76].

For instance, Sun et al. [77] studied human umbilical cord mesenchymal stem cell (hucMSC)-derived exosomes as a potential treatment for tissue repair after SCI. The authors reported that hucMSC-derived exosomes could effectively trigger the bone marrow-derived macrophage polarization from M1 to an M2 phenotype, down-regulate inflammatory cytokines, and enhance functional recovery after SCI.

Mu et al. [73] have also tackled the potential of MSC-derived exosomes. The researchers encapsulated these vesicles in fibrin glue that gelated in situ, providing in this manner a substrate for exosome delivery and nerve tissue growth. This treatment reduced inflammation and oxidative stress while promoting effective nerve tissue repair and functional recovery. In contrast, Li and colleagues [5] used a peptide-modified adhesive gel for exosome encapsulation, topical transplantation at the injured site, and sustained release in the host nerve tissues. This treatment resulted in considerable nerve recovery and urinary tissue preservation by efficiently mitigating inflammation and oxidation.

Differently, Luo et al. [74] have created a treatment based on M2-exosomes incorporated into a hydrogel. The exosomes stimulated vascular regeneration and functional recovery after SCI, as they induced a pro-angiogenic effect in spinal cord microvascular endothelial cells. Specifically, the high levels of ubiquitin thioesterase otulin from M2-exosomes are responsible for activating the Wnt/β-catenin signaling by increasing the protein level of β-catenin, positively modulating vascular regeneration and neurological functional recovery.

### 3.3. Cell Therapy

**Figure 4:**
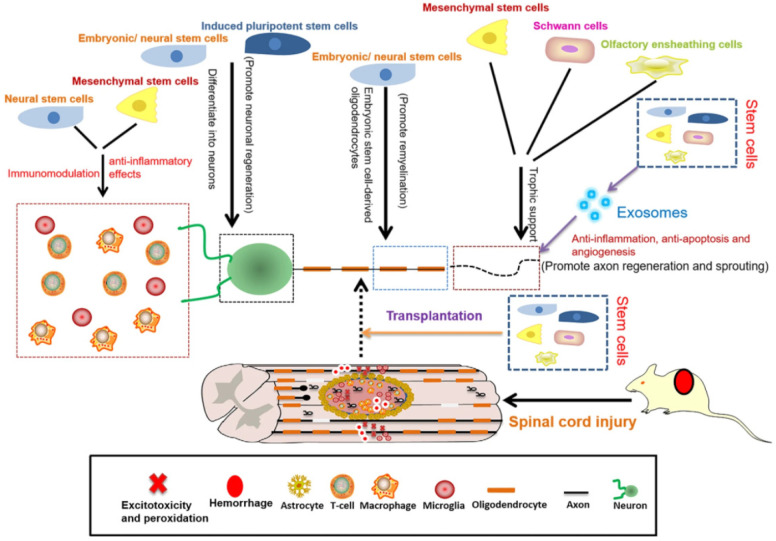
Schematic representation of the roles of stem cells in SCI repair. Reprinted from an open-access source [18].

SCI regeneration and repair can also be achieved through various cell therapies via two main methods: transplanting exogenous cells and directing or enhancing the functions of endogenous progenitor cells. The cell types studied in relation to spinal cord regeneration include embryonic, pluripotent, neural, and mesenchymal stem cells, oligodendrocyte and endothelial precursor cells, Schwann cells, olfactory ensheathing cells, and more [2,21,78] (Figure 4).

NSCs’ transplantation was especially studied in the spinal crush injury model, displaying encouraging results, such as differentiating into neurons, providing a neuronal substrate for electrical signals to bridge or circumvent the lesion area, accelerating axonal growth, and improving axonal conduction. Moreover, NSCs promote the survival and growth of damaged neurons to a series of secreted growth-promoting factors (e.g., BDNF, CNTF, GDNF, NGF, IGF-1) [79].

Important results have also been registered by grafting human spinal-cord-derived neural progenitor cells into cervical SCI sites of rhesus monkeys. Specifically, Rosenzweig et al. [80] reported that monkey axons regenerated into the NPC-based grafts and formed synapses, improving forelimb function. The encouraging outcomes led the authors to conclude that NPC graft therapy could be successful for reconstituting the human neural and glial milieu in the SCI site.

Another type of cell with great potential in SCI treatment is represented by embryonic stem cells (ESCs). ESCs can differentiate into neurons and glial cells that can reverse cell defects in the wounded area, while secreted active factors have the ability to impede further damage, support nerve tissue regeneration, achieve therapeutic effects, and improve motor dysfunction in tested animals [79].

Progress has been noted in the use of adipose-derived stem cells (ASCs). ASCs are considered promising therapeutic cells for SCI treatment, especially due to the plethora of molecules they secrete for modulating inflammatory response, stimulating axonal growth, promoting vascular remodeling, and ensuring cellular survival [3,81]. Thus, taking into account the therapeutic potential of ASCs, Pinho and colleagues [81] studied the administration of ASC secretome-based treatment on SCI mouse models. The authors reported this method as easy and reliable, with a positive effect on motor recovery and potential for application in models closer to humans as a means to refine it before translating to clinical trials.

An interesting emerging possibility for spinal cord regeneration is suggested by Rövekamp et al. [82]. The researchers exploited the olfactory mucosa as a source of multipotent cells. Specifically, the authors isolated, purified, and cultivated olfactory stem cells that further differentiated into the neural lineage. However, further research is required in the field, as there are no standard methods for purification, characterization, and delivery of olfactory stem cells to the injury site. These aspects necessitate clarification before clinical approval.

Numerous other studies have used cell therapy for spinal cord regeneration, yet researchers opted for delivering cells via biomaterial scaffolds instead of direct transplantation. Recent works of this kind are reviewed in Section 3.4.2 Cell-seeded scaffolds.

### 3.4. Biomaterial Scaffolds

In spinal cord regeneration, biomaterial scaffolds can be employed to refurbish the continuity of the injured site and ensure a suitable environment for tissue repair, axonal regeneration, and vascularization [83]. Both natural and synthetic biomaterials have been explored for designing adequate structures for restoring neurological function. Furthermore, the physicochemical properties of such biocompatible constructs can be engineered to allow tailored drug release and permit unobstructed space for cell growth and differentiation [8,35,84,85].

In particular, polymers have attracted special focus for designing biomaterial scaffolds due to their appropriate mechanical properties, fabrication versatility, and ability for multiple functionalization [8,85,86,87]. Thus, either alone or in composites, a broad range of natural (e.g., chitosan [84,88,89], alginate [2,90], agarose [91], gelatin [92,93], hyaluronic acid [94,95], collagen [83,84,96,97]) and synthetic (e.g., PLGA [98,99,100], PLA [101,102], PEG [103], polycaprolactone (PCL) [104,105,106], polysialic acid (PSA) [106]) polymers have been investigated for creating cell-free and cell-seeded scaffolds for spinal cord regeneration.

#### 3.4.1. Cell-Free Scaffolds

Numerous polymer-based cell-free scaffolds have been reported with different degrees of success in the literature. For instance, Ma and colleagues [107] have developed a decellularized spinal cord scaffold with a thin PLGA outer shell. The major inhibitory components were eliminated from the scaffold, creating a permissive matrix for integration and differentiation of NSCs into neurons. Moreover, the scaffold presented enhanced biocompatibility, suitable mechanical properties, and resilience to infiltration by myofibroblasts and the deposition of the dense collagen matrix. The authors also reported a mild immunogenic activity but a prominent ability to polarize macrophages from the M1 to the M2 phenotype. These aspects were further reflected in significant tissue regeneration and functional restoration after SCI.

On a different note, Qian et al. [108] have prepared a scaffold for the controlled release of melatonin for curing long-range nerve defects. In this respect, the researchers 3D-printed a melatonin/PCL nerve guide conduit that was able to enhance Schwann cell proliferation and neural expression towards stimulating functional, electrophysiological, and morphological in vivo regeneration of the nerves. In addition, the scaffold was noted to reduce oxidative stress, inflammation, mitochondrial dysfunction, and reduce nerve cell apoptosis, providing energy for nerves, facilitating nerve debris clearance, and stimulating neural proliferation.

Alternatively, Zhang et al. [106] utilized PCL in combination with PSA to create a hybrid nanofiber scaffold encapsulated with glucocorticoid methylprednisolone (MP). The authors reported that the PCL/PSA/MP scaffold could diminish the release of TNF-α and IL-6, decrease apoptosis-associated Caspase-3 protein expression, inhibit axonal demyelination and glial fibrillary acidic protein (GFAP) expression, and enhance neurofilament 200 (NF-200) expression, consequently promoting axonal growth and boosting functional recovery after SCI.

Encouraging results have also been published concerning various hydrogel-based cell-free scaffolds. For instance, Zhai et al. [109] used an interpenetrating network of diacrylated poly(ε-caprolactone)-b-poly(ethylene glycol)-b-poly(ε-caprolactone) triblock copolymer combined with RADA16 peptide pre-modified with a cell adhesive Arg-Gly-Asp sequence. The composite hydrogel retained the nanofibrous structure of the peptide, yet it underwent a much slower degradation, ensuring a sustained treatment. Moreover, the scaffold displayed excellent cytocompatibility, promoted differentiation of NSCs, and reduced cavitation, glial scar formation, and inflammation at the hemi-sectioned SCI model lesion sites in rats. Differently, Wang et al. [110] have recently developed a multifunctional nanocomposite hydrogel made from poly(citrate-maleic)-ε-polylysine (PME) and multi-walled carbon nanotubes. These scaffolds exhibited desirable properties, counting injectability, self-healing ability, tissue-adhesiveness, broad-spectrum antibacterial activity, UV-shielding performance, biomimetic mechanical modulus, and electroconductivity with spinal cord tissues, cytocompatibility, hemocompatibility, and biodegradability. Furthermore, in vivo tests revealed potential in enhancing locomotion recovery, reducing inflammation, promoting remyelination, and stimulating axon regeneration after SCI. Another hydrogel for SCI repair has been recently proposed by Shen and colleagues [111]. The authors created an immunoregulatory hydrogel that scavenges anionic damage-associated molecular patterns (DAMPs) and sustainedly releases IL-10 towards reducing the pro-inflammatory responses of macrophages and microglia, promoting the neurogenic differentiation of NSCs and ensuring axon growth formation without scar formation.

**Figure 5:**
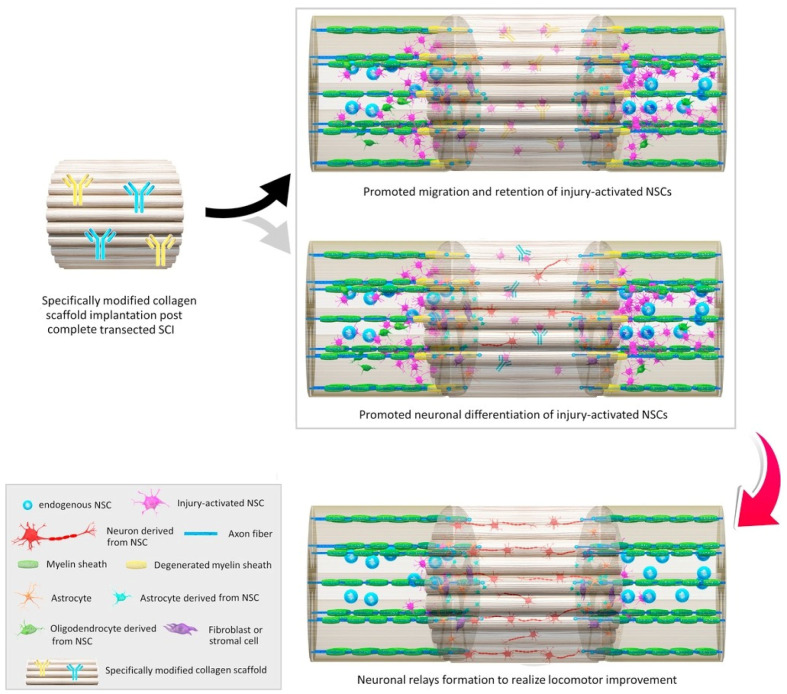
Schematic representation of in situ modulations of neuronal relay formation from injury-activated neural stem cells (NSCs) by implantation of functional bio-scaffolds in promoting locomotor outcome in animals after complete SCI. Reprinted from an open-access source [20].

Natural polymer-based scaffolds are considered equally or more suitable materials for tissue engineering and regeneration as an alternative to synthetic polymers. These natural materials present a series of advantageous properties, including biocompatibility, biodegradability, low immunogenicity, large surface area, similarity with extracellular matrix (ECM), tunable mechanical strength and conductivity, and easy large-scale production. Moreover, they are excellent support materials for living cells, small molecules, growth factors, and liposomes, ensuring their controlled and sustained release at the implantation site [2,112,113] (Figure 5).

For instance, Yeh and colleagues [114] fabricated a collagen scaffold for glial scar replacement. This polymeric structure could increase the expression of neurofilament and fibronectin while reducing the expression of glial fibrillary acidic protein and anti-chondroitin sulfate. These aspects were further reflected in enhanced neuronal survival and axonal growth, controlled astrocyte production, and prevention of glial scar formation.

Additionally tackling the benefits of collagen scaffolds, Yin et al. [115] have developed a linear-ordered collagen construct loaded with Taxol and implanted it in canine models after removing a portion of 1 cm from their spinal tissue. Observations during a half-year period revealed significantly increased neurogenesis and axon regeneration, reduced glial scar formation, and promoted motor-evoked potentials and locomotion recovery. Therefore, this method can be considered efficient for treating acute long-distance spinal cord defects.

Differently, Sun et al. [84] have created a collagen–chitosan hybrid scaffold that could partially re-establish a permissive microenvironment for axonal regeneration. The natural polymer-based scaffold decreased scar and cavity formation, enhanced nerve fiber regeneration, and improved functional recovery in rats, offering a promising therapeutic alternative for SCI.

In contrast, Han et al. [91] utilized another material. Specifically, the authors developed a Matrigel-loaded agarose scaffold that could support and improve linearly organized axon regeneration after SCI. Moreover, the scaffold was able to guide the reconnection of functional axons, contributing to locomotion recovery.

#### 3.4.2. Cell-Seeded Scaffolds

Incorporating various cells into biomaterial scaffolds is another promising regenerative approach for spinal cord repair, as several studies have reported encouraging outcomes.

One such example is offered by Li and colleagues [116], who have prepared a peptide-modified hyaluronic acid scaffold containing dispersed MnO_2_ nanoparticles and seeded with MSCs. The nanoparticles could alleviate the oxidative environment and improve MSCs viability, whereas the hydrogel enables adhesive growth of the seeded cells. The scaffold was noted to considerably restore motor function, induce in vivo integration and neural differentiation, and efficiently regenerate spinal cord tissue.

Alternatively, Yuan et al. [3] have fabricated a cell-adaptable neurogenic hydrogel seeded with ASCs. The as-designed scaffold provided an adequate matrix for cell infiltration, leading to enhanced axonal growth, improved motor-evoked potential, hindlimb strength, and complete spinal cord transection coordination. Moreover, this biomaterial structure was noted to induce macrophage polarization towards the M2 phenotype, suppressing neuroinflammation and cell apoptosis.

Kourgiantaki et al. [117] have created porous collagen-based scaffolds to deliver and protect embryonic NSCs at SCI sites. The authors reported a remarkable improvement in locomotion recovery, as the scaffolds could induce regeneration of injured regions through neural differentiation, functional integration, robust axonal elongation, and reduced astrogliosis. Similarly, Li and colleagues [118] have produced an NSCs-seeded collagen scaffold, but, in addition, they also loaded the material with paclitaxel-encapsulated liposomes. The as-designed scaffold ensured a prolonged sustained drug release at the lesion site while providing an instructive microenvironment for neuronal differentiation of NSCs, motor and sensory neuron regeneration, and axon extension. These processes are further conducted to enhance motor-evoked potential and hindlimb locomotion recovery.

You et al. [119] utilized bone mesenchymal stem cells (BMSCs) seeded on a porous silk fibroin scaffold to enhance transplanted cells’ survivability and promote nerve regeneration. The cell-seeded scaffold was noted to increase the markers for damaged axon regeneration and maintenance of the myelin structural and functional integrity, bridging the defected nerve with nerve fibers when applied to the transected spinal cord.

Another interesting regenerative strategy is proposed by Ham et al. [88], who used an NSC-seeded hydrogel scaffold with covalently immobilized interferon-γ and concomitantly administered intracellular σ peptide. Despite not being able to reconnect transplanted cells with the host tissue, this treatment resulted in an extension of neurofilament fibers from the host tissue into the scaffold and improved functional outcomes.

Alternatively, Lai and colleagues [120] have recently proposed the construction of niche-specific spinal white-matter-like tissue (WMLT) using decellularized optic nerves encapsulated with NT-3-overexpressing oligodendrocyte precursor cells. In the implanted structure, laminin was noted to promote the oligodendroglial lineage (OL) and guide linear axon regeneration via interactions with specific integrins on the axon surface. Thus, a niche rich in laminin, NT-3, and OL cells was created, which led to the considerable structural repair of SCI and significantly improved motor functions.

He and colleagues [78] have considered an innovative 3D-bioprinted scaffold composed of the neonatal acellular spinal cord and gelatin methacryloyl hydrogels seeded with menstrual-blood-derived mesenchymal stem cells (MenSCs). These components have the potential to work in synergy towards optimizing bioactive composition and microstructure, ensuring adequate mechanical properties, stimulating the spinal cord conduction path, and eventually leading to SCI rehabilitation.

### 3.5. Other Rehabilitation Strategies

**Figure 6:**
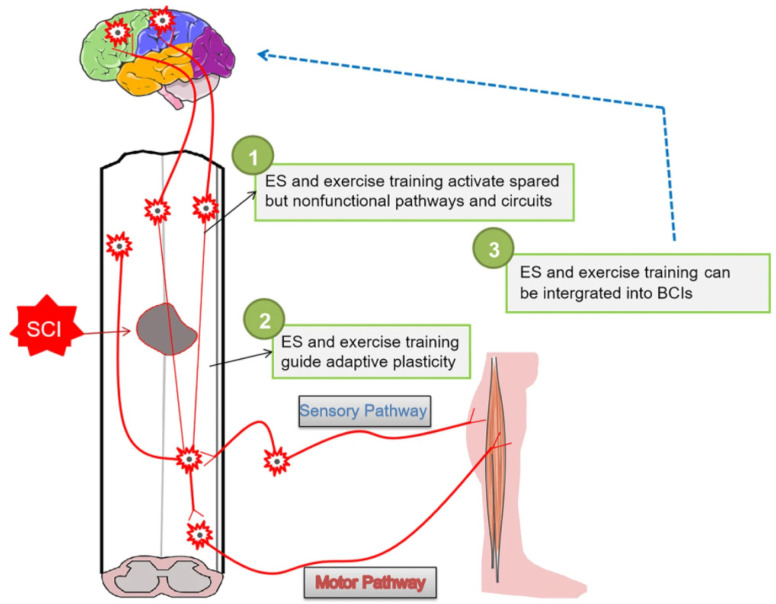
Schematic representation of rehabilitation strategies for the long-term recovery of effective neural circuits. Reprinted from an open-access source [18].

In addition to the above-presented spinal cord repair strategies, several other rehabilitation approaches have been recently reported in the literature, particularly based on electrostimulation (ES) (Figure 6).

One recent rehabilitation approach is offered by Lemos et al. [121], who have tested the Possover-LION procedure in patients with chronic SCI. The laparoscopic implantation of neuromodulation electrodes was reported to improve the mobility and genital sensitivity of the patients, while also reducing the number of urinary and fecal incontinence episodes. Thus, the authors concluded that this procedure should be considered a safe and efficient therapy for patients with chronic SCI.

Similarly, Adeel et al. [122] investigated paired stimulation in subjects with chronic SCI. By testing different waveforms, the researchers observed that rTMS-iTBS/tsDCS and rTMS-20 Hz/tsDCS improved motor-evoked potential (MEP) latency, MEP amplitude, and lower extremity motor scale during a single neuromodulation experimental trial.

An interesting therapeutic alternative is also offered by Olmsted and colleagues [123]. The authors prepared alginate-based neural ribbons of synaptically connected neuronal networks composed of functionally maturing caudal spinal motor neurons, interneurons, and oligodendrocyte progenitor cells. The researchers directed neurite formation within these ribbons toward generating electrically active, synaptically connected networks. These structures were tested in vivo, exhibiting viability and retention of interconnected synaptic networks that readily integrate with the host parenchyma. Therefore, transplantable neural circuitry for SCI treatment holds great promise for SCI treatment.

Recent research also focused on improving spinal-cord-related clinical procedures to enhance repair outcomes. In this respect, Kubelick and Emelianov [124] have developed a combined ultrasound (US)–photoacoustic (PA)–magnetic resonance (MR) imaging approach enhanced by Prussian blue nanocubes. (PBNCs). Specifically, the scientists acquired US/PA images while directly injecting PBNC-labeled stem cells into the spinal cord, whereas US/PA/MR images were acquired post-surgery. This imaging approach allowed the multimodal detection of low concentrations of stem cells, real-time needle and injection guidance, and immediate feedback on stem cell delivery, being an auspicious strategy to guide stem cell injections intraoperatively and monitor stem cell therapies in the spinal cord postoperatively.

## 4. Conclusions

As the spinal cord cannot regenerate itself and no treatment is yet clinically available for its complete healing, spinal cord injury remains one of the most devastating diseases, affecting the quality of life of millions of patients and imposing an economic burden on healthcare systems worldwide.

Considering the impetuous need for developing efficient treatment strategies, scientists have recently approached SCIs from an interdisciplinary perspective. Thus, a series of promising discoveries concerning biomolecules and stem cells mechanisms in the SCI microenvironment have been reported in the literature, while tremendous progress has been noticed in designing various drug delivery vehicles and biomaterial scaffolds. However, most of the studies have been conducted in vitro and/or in vivo on small animals; only two of the cited studies were performed on larger animals (i.e., rhesus monkey, canine models), while none of the potential therapies have reached the clinical testing stage. There are a number of challenges and limitations associated with translating these advanced therapeutic strategies, such as improperly powered studies with insufficient animal numbers, heterogeneity of SCI models, and interspecies variations in neuroanatomy. Moreover, factors such as animal age, inbreeding, and housing may also represent obstacles in clinical translation, as humans have very different immune systems and behaviors than inbred mouse strains living in a sterile environment; hence, the neuroplasticity, regeneration, and recovery after an SCI is also expected to be different. More specifically, before moving into the clinic, the newly developed delivery nanosystems must be thoroughly investigated from the points of view of their cytotoxicity (focusing on their overall effects on human health rather than on specific cells and tissues) and their ability to translate from laboratory to scaled-up synthesis without affecting their quality. The advanced repair strategies involving different biomolecules and stem cells require further research to fully understand these entities’ mechanisms of action and their long-term effects. On the other hand, the main challenge in using biomaterial scaffolds resides in finding the balance between adequate physicochemical properties and maximum therapeutic efficacy.

To conclude, encouraging results have been obtained by testing a wide range of therapeutic options for accomplishing spinal cord regeneration and repair, yet further research is required concerning their effects in human use before these strategies can be implemented in clinical practice.

## Figures and Tables

**Table 1:** Comparison between primary and secondary spinal cord injury (SCI). Created based on information from [4,18,20,21,22,23].

Injury Type	Primary Injury	Secondary Injury
Comparison Criteria		
Description	Mechanical disruption of the spinal cord during trauma	Rapidly escalating cascade of acute and chronic degenerative events
Cause	External force acting directly or indirectly on the spinal cord	Chemical and physical events created by the primary injury
Characteristics	Mechanical destruction of neural tissue Hemorrhage within the spinal cord Compression Laceration Transection	Oxidative stress Loss of mitochondrial homeostasis Ischemia Hypoxia Cellular damage Demyelination of axons Glial scar formation Disconnection of living neurons Inflammation Immune response

**Table 2:** Examples of metal-based nanosystems for spinal cord delivery.

Nanocarrier Material	Cargo	Characteristics	Refs.
Gold	Zonisamide	Morphology: nanospheres Average size without loading: 14 nm Average size with loading: 43.0 ± 2.2. nm	[42]
Gold	Herbal medicines	Morphology: nanoclusters Average size <3 nm	[34]
Gold (conjugated with β-cyclodextrin)	Ursodeoxycholic acid	Morphology: nanospheres Average size without loading: 20–30 nm Average size with loading: 20–40 nm	[43]
Gold (caped with glutathione)	-	Morphology: spherical nanodots Average size: 2.3–2.8 nm	[36]
Maghemite (modified with PEG)	Tacrolimus	Morphology: nanospheres	[45]

**Table 3:** Examples of polymer-based nanosystems for spinal cord delivery.

Nanocarrier Material	Cargo	Characteristics	Refs.
PLGA	ChABC enzyme	Morphology: nanospheres Average size: 273.5 ± 36.4 nm	[49]
PLGA	Superoxide dismutase Catalase	Morphology: circular structures Average size: 122 ± 5.5 nm	[23]
PLA	Docosahexaenoic acid	Morphology: core-shell nanofibers Average core diameter: 300 nm Average shell thickness: 80 nm	[50]
PEG/PPG/PEG	Zonisamide	Morphology: almost spherical nanomicelles Average size: 105 nm	[51]
Stearic acid-chitosan	Sesamol	Morphology: spherical nanomicelles Hydrodynamic radius without loading: 53.12 ± 6.21 nm Hydrodynamic radius with loading: 59.12 ± 7.31 nm	[52]
Chitosan	CeO_2_ nanoparticles	Morphology: core-shell nanospheres Average size: 15–25 nm	[53]

**Table 4:** Examples of liposomal nanosystems for spinal cord delivery.

Nanocarrier Material	Cargo	Characteristics	Refs.
Cationic liposomes with vitamin E succinate-grafted ε-polylysine	pOXR1	Morphology: self-assembled micelles Average size of empty nanoparticles: 20 nm Size of pOXR1 after compression: 58 nm	[56]
Macrophage membrane-camouflaged liposomes	Minocycline	Morphology: core-shell nanostructure Average size: 110.08 ± 1.97 nm	[57]
Liposome covered by a chitosan oligosaccharide lactate layer	Neuropeptide apamin Curcumin	Morphology: nanospheres Average size without coating: 103.5 nm Average size with coating: 122.5 nm	[58]

**Table 5:** Examples of other types of nanosystems for spinal cord delivery.

Nanocarrier	Cargo	Characteristics	Refs.
Polyehtylene glycol-polyethylenimine (PEG-PEI) nanogel	Rolipram	Morphology: colloidal dispersion	[60]
Macrophage-derived cell membranes (modified with glutathione)	Metformin nanogel	Morphology: core-shell nanostructure Average size of nanogel core: 141.26 ± 5.70 nm Average size of the final structure: 148.03 ± 7.10 nm	[22]
Alginate–chitosan hydrogel	Berberine-encapsulated chitosan nanoparticles	Average size of chitosan nanoparticles without loading: 214 ± 42 nm Average size of chitosan nanoparticles with loading: ~252 nm	[61]
Inverse opal film	BPQDs FGF10 Chloroquine phosphate	Morphology: periodic hexagonal close-packed structure (prior to stretching)	[62]
Nanovesicles derived from macrophage membrane	Sodium alginate Naloxone	Morphology: nanospheres Average size without loading: 80 ± 12 nm Average size with sodium alginate: 112 ± 8 nm Average size with sodium alginate and naloxone: 134 ± 11 nm	[63]

## Data Availability

Not applicable.

## References

[1] Hong Q., Song H., Lan Chi N.T., Brindhadevi K. (2022). Numerous nanoparticles as drug delivery system to control secondary immune response and promote spinal cord injury regeneration. Process Biochem..

[2] Grijalvo S., Nieto-Díaz M., Maza R.M., Eritja R., Díaz D.D. (2019). Alginate Hydrogels as Scaffolds and Delivery Systems to Repair the Damaged Spinal Cord. Biotechnol. J..

[3] Yuan X., Yuan W., Ding L., Shi M., Luo L., Wan Y., Oh J., Zhou Y., Bian L., Deng D.Y.B. (2021). Cell-adaptable dynamic hydrogel reinforced with stem cells improves the functional repair of spinal cord injury by alleviating neuroinflammation. Biomaterials.

[4] Papa S., Mauri E., Rossi F., Perale G., Veglianese P., Perale G., Rossi F. (2020). Chapter 1—Introduction to spinal cord injury as clinical pathology. Spinal Cord Injury (SCI) Repair Strategies.

[5] Li L., Zhang Y., Mu J., Chen J., Zhang C., Cao H., Gao J. (2020). Transplantation of Human Mesenchymal Stem-Cell-Derived Exosomes Immobilized in an Adhesive Hydrogel for Effective Treatment of Spinal Cord Injury. Nano Lett..

[6] Benech C.A., Tomatis A., Perez R., Boido B., Perale G., Rossi F. (2020). Chapter 3—Conventional treatments and surgical margins of maneuvering for spinal cord injury management. Spinal Cord Injury (SCI) Repair Strategies.

[7] Squair J.W., Gautier M., Sofroniew M.V., Courtine G., Anderson M.A. (2021). Engineering spinal cord repair. Curr. Opin. Biotechnol..

[8] Luo Y., Xue F., Liu K., Li B., Fu C., Ding J. (2021). Physical and biological engineering of polymer scaffolds to potentiate repair of spinal cord injury. Mater. Des..

[9] Nas K., Yazmalar L., Şah V., Aydın A., Öneş K. (2015). Rehabilitation of spinal cord injuries. World J. Orthop..

[10] Rouanet C., Reges D., Rocha E., Gagliardi V., Silva G.S. (2017). Traumatic spinal cord injury: Current concepts and treatment update. Arq. Neuro. Psiquiatr..

[11] Chen X., Wang Y., Zhou G., Hu X., Han S., Gao J. (2021). The combination of nanoscaffolds and stem cell transplantation: Paving a promising road for spinal cord injury regeneration. Biomed. Pharmacother..

[12] Zimmermann R., Vieira Alves Y., Sperling L.E., Pranke P. (2020). Nanotechnology for the Treatment of Spinal Cord Injury. Tissue Eng. Part B Rev..

[13] Lowe T.L., Agrahari V., Kannan R.M., Kannan S. (2019). Nanotechnology enabled regenerative medicine for neurological disorders. Adv. Drug Deliv. Rev..

[14] Khan T.I., Hemalatha S., Waseem M. (2020). Promising Role of Nano-Encapsulated Drugs for Spinal Cord Injury. Mol. Neurobiol..

[15] Kiyotake E.A., Martin M.D., Detamore M.S. (2022). Regenerative rehabilitation with conductive biomaterials for spinal cord injury. Acta Biomater..

[16] Papa S., Pizzetti F., Perale G., Veglianese P., Rossi F. (2020). Regenerative medicine for spinal cord injury: Focus on stem cells and biomaterials. Expert Opin. Biol. Ther..

[17] Kaplan B., Levenberg S. (2022). The Role of Biomaterials in Peripheral Nerve and Spinal Cord Injury: A Review. Int. J. Mol. Sci..

[18] Yang B., Zhang F., Cheng F., Ying L., Wang C., Shi K., Wang J., Xia K., Gong Z., Huang X. (2020). Strategies and prospects of effective neural circuits reconstruction after spinal cord injury. Cell Death Dis..

[19] Quaye M., Harvey J., Ambrosio L., Tanner E. (2012). 3—Introduction to spinal pathologies and clinical problems of the spine. Biomaterials for Spinal Surgery.

[20] Li X., Liu D., Xiao Z., Zhao Y., Han S., Chen B., Dai J. (2019). Scaffold-facilitated locomotor improvement post complete spinal cord injury: Motor axon regeneration versus endogenous neuronal relay formation. Biomaterials.

[21] Saremi J., Mahmoodi N., Rasouli M., Ranjbar F.E., Mazaheri E.L., Akbari M., Hasanzadeh E., Azami M. (2022). Advanced approaches to regenerate spinal cord injury: The development of cell and tissue engineering therapy and combinational treatments. Biomed. Pharmacother..

[22] Yu Q., Jiang X., Liu X., Shen W., Mei X., Tian H., Wu C. (2022). Glutathione-modified macrophage-derived cell membranes encapsulated metformin nanogels for the treatment of spinal cord injury. Mater. Sci. Eng. C.

[23] Andrabi S.S., Yang J., Gao Y., Kuang Y., Labhasetwar V. (2020). Nanoparticles with antioxidant enzymes protect injured spinal cord from neuronal cell apoptosis by attenuating mitochondrial dysfunction. J. Control. Release.

[24] Ferrero B., Di Liberto A., Perale G., Rossi F. (2020). Chapter 2—Spinal cord injury: Role of neurophysiology. Spinal Cord Injury (SCI) Repair Strategies.

[25] Rupp R., Biering-Sørensen F., Burns S.P., Graves D.E., Guest J., Jones L., Read M.S., Rodriguez G.M., Schuld C., Tansey-Md K.E. (2021). International standards for neurological classification of spinal cord injury: Revised 2019. Top. Spinal Cord Inj. Rehab..

[26] Baptiste D.C., Fehlings M.G., Weber J.T., Maas A.I.R. (2007). Update on the treatment of spinal cord injury. Progress in Brain Research.

[27] Khadka B., Lee J.-Y., Kim K.-T., Bae J.-S. (2020). Recent progress in therapeutic drug delivery systems for treatment of traumatic CNS injuries. Future Med. Chem..

[28] Pandit R., Chen L., Götz J. (2020). The blood-brain barrier: Physiology and strategies for drug delivery. Adv. Drug Deliv. Rev..

[29] Monajjemi M., Mollaamin F. (2020). Bio-capacitor consist of insulated myelin-sheath and uninsulated node of Ranvier: A bio-nano-antenna. Biointerface Res. Appl. Chem..

[30] Jin L.-Y., Li J., Wang K.-F., Xia W.-W., Zhu Z.-Q., Wang C.-R., Li X.-F., Liu H.-Y. (2020). Blood–Spinal Cord Barrier in Spinal Cord Injury: A Review. J. Neurotrauma.

[31] Montague-Cardoso K., Malcangio M. (2021). Changes in blood–spinal cord barrier permeability and neuroimmune interactions in the underlying mechanisms of chronic pain. Pain Rep..

[32] Cross C.G., Payne A.H., Hawryluk G.W., Haag-Roeger R., Cheeniyil R., Brady D., Odéen H., Minoshima S., Cross D.J., Anzai Y. (2021). Technical Note: Quantification of blood-spinal cord barrier permeability after application of magnetic resonance-guided focused ultrasound in spinal cord injury. Med. Phys..

[33] Wang J., Nie Z., Zhao H., Gao K., Cao Y. (2020). MiRNA-125a-5p attenuates blood–spinal cord barrier permeability under hypoxia in vitro. Biotechnol. Lett..

[34] Zhou Z., Li D., Fan X., Lin S., Yuan Y., Zhuang P., Hu H., Ge M., Chen S., Mei X. (2022). Gold nanoclusters for optimizing the general efficacies of herbal medicines on nerve repair after spinal cord injury. Mater. Des..

[35] Mauri E., Masi M., Perale G., Rossi F. (2020). Chapter 8—Nanomaterials for spinal cord injury (SCI) regeneration. Spinal Cord Injury (SCI) Repair Strategies.

[36] Lin Y., Zhao Y., Yang Z., Shen Z., Ke J., Yin F., Fang L., Zvyagin A.V., Yang B., Lin Q. (2022). Gold nanodots with stable red fluorescence for rapid dual-mode imaging of spinal cord and injury monitoring. Talanta.

[37] Bayoumy A.M., Elhaes H., Osman O., Hussein T., Ibrahim M.A. (2020). Mapping molecular electrostatic potential for heme interacting with nano metal oxides. Biointerface Res. Appl. Chem..

[38] Bayoumy A.M., Elhaes H., Osman O., Kholmurodov K.T., Hussein T., Ibrahim M.A. (2020). Effect of nano metal oxides on heme molecule: Molecular and biomolecular approaches. Biointerface Res. Appl. Chem..

[39] Eivazzadeh-Keihan R., Bahojb Noruzi E., Khanmohammadi Chenab K., Jafari A., Radinekiyan F., Hashemi S.M., Ahmadpour F., Behboudi A., Mosafer J., Mokhtarzadeh A. (2020). Metal-based nanoparticles for bone tissue engineering. J. Tissue Eng. Regen. Med..

[40] Aghaie T., Jazayeri M.H., Manian M., Khani L., Erfani M., Rezayi M., Ferns G.A., Avan A. (2019). Gold nanoparticle and polyethylene glycol in neural regeneration in the treatment of neurodegenerative diseases. J. Cell. Biochem..

[41] Zhang J., Mou L., Jiang X. (2020). Surface chemistry of gold nanoparticles for health-related applications. Chem. Sci..

[42] Fang C., Ma Z., Chen L., Li H., Jiang C., Zhang W. (2019). Biosynthesis of gold nanoparticles, characterization and their loading with zonisamide as a novel drug delivery system for the treatment of acute spinal cord injury. J. Photochem. Photobiol. B Biol..

[43] Kim S.J., Ko W.-K., Heo D.N., Lee S.J., Lee D., Heo M., Han I.-B., Kwon I.K., Sohn S. (2019). Anti-neuroinflammatory gold nanocomplex loading ursodeoxycholic acid following spinal cord injury. Chem. Eng. J..

[44] Renitta R.E., Smitha I., Sahithya C.S., Samrot A.V., Abirami S., Dhiva S., Anand D.A. (2021). Synthesis, Characterization, and Antibacterial Activity of Biosynthesized Gold Nanoparticles. Biointerface Res. Appl. Chem..

[45] Wang J., Xie T., Long X., Gao R., Kang L., Wang Q., Jiang J., Ye L., Lyu J. (2022). The effect of tacrolimus-containing polyethylene glycol-modified maghemite nanospheres on reducing oxidative stress and accelerating the healing spinal cord injury of rats based on increasing M2 macrophages. Arab. J. Chem..

[46] Guo S., Fu D., Utupova A., Sun D., Zhou M., Jin Z., Zhao K. (2019). Applications of polymer-based nanoparticles in vaccine field. Nanotechnol. Rev..

[47] Liang J., Zhao X. (2021). Nanomaterial-based delivery vehicles for therapeutic cancer vaccine development. Cancer Biol. Med..

[48] Zielińska A., Carreiró F., Oliveira A.M., Neves A., Pires B., Venkatesh D.N., Durazzo A., Lucarini M., Eder P., Silva A.M. (2020). Polymeric Nanoparticles: Production, Characterization, Toxicology and Ecotoxicology. Molecules.

[49] Azizi M., Farahmandghavi F., Joghataei M.T., Zandi M., Imani M., Bakhtiari M., Omidian H. (2020). ChABC-loaded PLGA nanoparticles: A comprehensive study on biocompatibility, functional recovery, and axonal regeneration in animal model of spinal cord injury. Int. J. Pharm..

[50] Liu Z.-H., Huang Y.-C., Kuo C.-Y., Kuo C.-Y., Chin C.-Y., Yip P.K., Chen J.-P. (2020). Docosahexaenoic Acid-Loaded Polylactic Acid Core-Shell Nanofiber Membranes for Regenerative Medicine after Spinal Cord Injury: In Vitro and In Vivo Study. Int. J. Mol. Sci..

[51] Nie S., Lu J., Huang Y., Li Q.-a. (2021). Zonisamide-loaded triblock copolymer nanomicelle as a controlled drug release platform for the treatment of oxidative stress -induced spinal cord neuronal damage. J. Mol. Liq..

[52] Wang N., Yu H., Song Q., Mao P., Li K., Bao G. (2021). Sesamol-loaded stearic acid-chitosan nanomicelles mitigate the oxidative stress-stimulated apoptosis and induction of pro-inflammatory cytokines in motor neuronal of the spinal cord through NF-ĸB signaling pathway. Int. J. Biol. Macromol..

[53] Fang X., Song H. (2019). Synthesis of cerium oxide nanoparticles loaded on chitosan for enhanced auto-catalytic regenerative ability and biocompatibility for the spinal cord injury repair. J. Photochem. Photobiol. B Biol..

[54] Large D.E., Abdelmessih R.G., Fink E.A., Auguste D.T. (2021). Liposome composition in drug delivery design, synthesis, characterization, and clinical application. Adv. Drug Deliv. Rev..

[55] Guimarães D., Cavaco-Paulo A., Nogueira E. (2021). Design of liposomes as drug delivery system for therapeutic applications. Int. J. Pharm..

[56] Zhang J., Li Y., Xiong J., Xu H., Xiang G., Fan M., Zhou K., Lin Y., Chen X., Xie L. (2021). Delivery of pOXR1 through an injectable liposomal nanoparticle enhances spinal cord injury regeneration by alleviating oxidative stress. Bioact. Mater..

[57] Tang W., Yang Y., Yang L., Tang M., Chen Y., Li C. (2021). Macrophage membrane-mediated targeted drug delivery for treatment of spinal cord injury regardless of the macrophage polarization states. Asian J. Pharm. Sci..

[58] Wang X., Wu J., Liu X., Tang K., Cheng L., Li J., Tang Y., Song X., Wang X., Li C. (2021). Engineered liposomes targeting the gut–CNS Axis for comprehensive therapy of spinal cord injury. J. Control. Release.

[59] Vashist A., Kaushik A., Vashist A., Bala J., Nikkhah-Moshaie R., Sagar V., Nair M. (2018). Nanogels as potential drug nanocarriers for CNS drug delivery. Drug Discov. Today.

[60] Papa S., Veneruso V., Mauri E., Cremonesi G., Mingaj X., Mariani A., de Paola M., Rossetti A., Sacchetti A., Rossi F. (2021). Functionalized nanogel for treating activated astrocytes in spinal cord injury. J. Control. Release.

[61] Mahya S., Ai J., Shojae S., Khonakdar H.A., Darbemamieh G., Shirian S. (2021). Berberine loaded chitosan nanoparticles encapsulated in polysaccharide-based hydrogel for the repair of spinal cord. Int. J. Biol. Macromol..

[62] Wu F., Zu Y., Weng W., Yang Y., Hu J., Mao Y., Shao C., Xiao J. (2022). Multifunctional inverse opal film as a responsive drug carrier for spinal cord injury repair. Chem. Eng. J..

[63] Liu X., Jiang X., Yu Q., Shen W., Tian H., Mei X., Wu C. (2022). Sodium alginate and naloxone loaded macrophage-derived nanovesicles for the treatment of spinal cord injury. Asian J. Pharm. Sci..

[64] Ashammakhi N., Kim H.-J., Ehsanipour A., Bierman R.D., Kaarela O., Xue C., Khademhosseini A., Seidlits S.K. (2019). Regenerative Therapies for Spinal Cord Injury. Tissue Eng. Part B Rev..

[65] Oudega M., Hao P., Shang J., Haggerty A.E., Wang Z., Sun J., Liebl D.J., Shi Y., Cheng L., Duan H. (2019). Validation study of neurotrophin-3-releasing chitosan facilitation of neural tissue generation in the severely injured adult rat spinal cord. Exp. Neurol..

[66] Cong Y., Wang C., Wang J., Li H., Li Q. (2020). NT-3 Promotes Oligodendrocyte Proliferation and Nerve Function Recovery After Spinal Cord Injury by Inhibiting Autophagy Pathway. J. Surg. Res..

[67] Afrash H., Nazeri N., Davoudi P., FaridiMajidi R., Ghanbari H. (2021). Development of a Bioactive Scaffold based on NGF Containing PCL/Chitosan Nanofibers for Nerve Regeneration. Biointerface Res. Appl. Chem..

[68] Zyuz’kov G.N., Stavrova L.A., Miroshnichenko L.A., Polykova T.Y., Simanina E.V. (2021). Prospects for the Use of NF-kappa b Inhibitors to Stimulate the Functions of Regeneration-Competent Cells of Nerve Tissue and Neuroregeneration in Ethanol-Induced Neurodegeneration. Biointerface Res. Appl. Chem..

[69] Yamanaka K., Eldeiry M., Aftab M., Ryan T.J., Meng X., Weyant M.J., Fullerton D.A., Reece T.B. (2019). Synergetic Induction of NGF With Diazoxide and Erythropoietin Attenuates Spinal Cord Ischemic Injury. J. Surg. Res..

[70] Cheng Y.-Y., Zhao H.-K., Chen L.-W., Yao X.-Y., Wang Y.-L., Huang Z.-W., Li G.-P., Wang Z., Chen B.-Y. (2020). Reactive astrocytes increase expression of proNGF in the mouse model of contused spinal cord injury. Neurosci. Res..

[71] Li J.-Y., Liu J., Manaph N.P.A., Bobrovskaya L., Zhou X.-F. (2017). ProBDNF inhibits proliferation, migration and differentiation of mouse neural stem cells. Brain Res..

[72] Bahardoust M., Baghoi-Hosseinabadi Z. (2021). Role of Adipose-Derived Mesenchymal Stem Cells in the Regeneration of Cardiac Tissue and Improvement of Cardiac Function: A Narrative Review. Biointerface Res. Appl. Chem..

[73] Mu J., Wu J., Cao J., Ma T., Li L., Feng S., Gao J. (2021). Rapid and effective treatment of traumatic spinal cord injury using stem cell derived exosomes. Asian J. Pharm. Sci..

[74] Luo Z., Peng W., Xu Y., Xie Y., Liu Y., Lu H., Cao Y., Hu J. (2021). Exosomal OTULIN from M2 macrophages promotes the recovery of spinal cord injuries via stimulating Wnt/β-catenin pathway-mediated vascular regeneration. Acta Biomater..

[75] Hu Q., Lyon C.J., Fletcher J.K., Tang W., Wan M., Hu T.Y. (2021). Extracellular vesicle activities regulating macrophage- and tissue-mediated injury and repair responses. Acta Pharm. Sin. B.

[76] Khalatbary A.R. (2021). Stem cell-derived exosomes as a cell free therapy against spinal cord injury. Tissue Cell.

[77] Sun G., Li G., Li D., Huang W., Zhang R., Zhang H., Duan Y., Wang B. (2018). hucMSC derived exosomes promote functional recovery in spinal cord injury mice via attenuating inflammation. Mater. Sci. Eng. C.

[78] He W., Ju D., Gu Y., Lu Y., Ge M., Wu Q., Dong C. (2022). Human menstrual blood-derived stem cells combined with a new 3D bioprinted composite scaffold for spinal cord injury treatment. Med. Hypotheses.

[79] Shao A., Tu S., Lu J., Zhang J. (2019). Crosstalk between stem cell and spinal cord injury: Pathophysiology and treatment strategies. Stem Cell Res. Ther..

[80] Rosenzweig E.S., Brock J.H., Lu P., Kumamaru H., Salegio E.A., Kadoya K., Weber J.L., Liang J.J., Moseanko R., Hawbecker S. (2018). Restorative effects of human neural stem cell grafts on the primate spinal cord. Nat. Med..

[81] Pinho A.G., Cibrão J.R., Lima R., Gomes E.D., Serra S.C., Lentilhas-Graça J., Ribeiro C., Lanceros-Mendez S., Teixeira F.G., Monteiro S. (2022). Immunomodulatory and regenerative effects of the full and fractioned adipose tissue derived stem cells secretome in spinal cord injury. Exp. Neurol..

[82] Rövekamp M., von Glinski A., Volkenstein S., Dazert S., Sengstock C., Schildhauer T.A., Breisch M. (2022). Olfactory Stem Cells for the Treatment of Spinal Cord Injury—A New Pathway to the Cure?. World Neurosurg..

[83] Mneimneh A.T., Mehanna M.M. (2021). Collagen-based scaffolds: An auspicious tool to support repair, recovery, and regeneration post spinal cord injury. Int. J. Pharm..

[84] Sun Y., Yang C., Zhu X., Wang J.-J., Liu X.-Y., Yang X.-P., An X.-W., Liang J., Dong H.-J., Jiang W. (2019). 3D printing collagen/chitosan scaffold ameliorated axon regeneration and neurological recovery after spinal cord injury. J. Biomed. Mater. Res. Part A.

[85] Zhang Q., Shi B., Ding J., Yan L., Thawani J.P., Fu C., Chen X. (2019). Polymer scaffolds facilitate spinal cord injury repair. Acta Biomater..

[86] Gloria A., De Santis R., Ambrosio L., Tanner K.E., Ambrosio L., Tanner E. (2012). 8—Artificial intervertebral discs. Biomaterials for Spinal Surgery.

[87] Raucci M.G., Gloria A., de Santis R., Ambrosio L., Tanner K.E., Ambrosio L., Tanner E. (2012). 1—Introduction to biomaterials for spinal surgery. Biomaterials for Spinal Surgery.

[88] Ham T.R., Pukale D.D., Hamrangsekachaee M., Leipzig N.D. (2020). Subcutaneous priming of protein-functionalized chitosan scaffolds improves function following spinal cord injury. Mater. Sci. Eng. C.

[89] Kurakula M., Gorityala S., Patel D.B., Basim P., Patel B., Kumar Jha S. (2021). Trends of Chitosan Based Delivery Systems in Neuroregeneration and Functional Recovery in Spinal Cord Injuries. Polysaccharides.

[90] Jahandideh A., Noori H., Rahimi B., Hamblin M.R., Behroozi Z., Ramezani M., Ramezani F. (2021). Alginate scaffolds improve functional recovery after spinal cord injury. Eur. J. Trauma Emerg. Surg..

[91] Han S., Lee J.Y., Heo E.Y., Kwon I.K., Yune T.Y., Youn I. (2018). Implantation of a Matrigel-loaded agarose scaffold promotes functional regeneration of axons after spinal cord injury in rat. Biochem. Biophys. Res. Commun..

[92] Babaloo H., Ebrahimi-Barough S., Derakhshan M.A., Yazdankhah M., Lotfibakhshaiesh N., Soleimani M., Joghataei M.-T., Ai J. (2019). PCL/gelatin nanofibrous scaffolds with human endometrial stem cells/Schwann cells facilitate axon regeneration in spinal cord injury. J. Cell. Physiol..

[93] Fan L., Liu C., Chen X., Zou Y., Zhou Z., Lin C., Tan G., Zhou L., Ning C., Wang Q. (2018). Directing Induced Pluripotent Stem Cell Derived Neural Stem Cell Fate with a Three-Dimensional Biomimetic Hydrogel for Spinal Cord Injury Repair. ACS Appl. Mater. Interfaces.

[94] Elkhenany H., Bonilla P., Giraldo E., Alastrue Agudo A., Edel M.J., Vicent M.J., Roca F.G., Ramos C.M., Doblado L.R., Pradas M.M. (2021). A Hyaluronic Acid Demilune Scaffold and Polypyrrole-Coated Fibers Carrying Embedded Human Neural Precursor Cells and Curcumin for Surface Capping of Spinal Cord Injuries. Biomedicines.

[95] Jensen G., Holloway J.L., Stabenfeldt S.E. (2020). Hyaluronic Acid Biomaterials for Central Nervous System Regenerative Medicine. Cells.

[96] Zou Y., Ma D., Shen H., Zhao Y., Xu B., Fan Y., Sun Z., Chen B., Xue W., Shi Y. (2020). Aligned collagen scaffold combination with human spinal cord-derived neural stem cells to improve spinal cord injury repair. Biomater. Sci..

[97] Deng W.-S., Ma K., Liang B., Liu X.-Y., Xu H.-Y., Zhang J., Shi H.-Y., Sun H.-T., Chen X.-Y., Zhang S. (2020). Collagen scaffold combined with human umbilical cord-mesenchymal stem cells transplantation for acute complete spinal cord injury. Neural. Regen. Res..

[98] Han I.-B., Thakor D.K., Ropper A.E., Yu D., Wang L., Kabatas S., Zeng X., Kim S.-W., Zafonte R.D., Teng Y.D. (2019). Physical impacts of PLGA scaffolding on hMSCs: Recovery neurobiology insight for implant design to treat spinal cord injury. Exp. Neurol..

[99] Kong W., Qi Z., Xia P., Chang Y., Li H., Qu Y., Pan S., Yang X. (2019). Local delivery of FTY720 and NSCs on electrospun PLGA scaffolds improves functional recovery after spinal cord injury. RSC Adv..

[100] Pan S., Zhao Y., Qiao X., Qi Z., Fu C., Kong W., Liu Q., Yang X. (2019). PLGA porous scaffolds by polydopamine-assisted immobilization of NGF for spinal cord injury repair. Mater. Res. Express.

[101] Biagini G., Marcon B.H., Pereira T., Berti L.F., Senegaglia A.C., Correa A., Stimamiglio M.A. (2021). Analysis of the Interaction Between Stem Cells and Polylactic Acid (Pla) Scaffolds For Applications In Tissue Engineering. Cytotherapy.

[102] Raynald, Shu B., Liu X.-B., Zhou J.-F., Huang H., Wang J.-Y., Sun X.-D., Qin C., An Y.-H. (2019). Polypyrrole/polylactic acid nanofibrous scaffold cotransplanted with bone marrow stromal cells promotes the functional recovery of spinal cord injury in rats. CNS Neurosci. Ther..

[103] Lu X., Perera T.H., Aria A.B., Callahan L.A.S. (2018). Polyethylene glycol in spinal cord injury repair: A critical review. J. Exp. Pharmacol..

[104] Zhou X., Shi G., Fan B., Cheng X., Zhang X., Wang X., Liu S., Hao Y., Wei Z., Wang L. (2018). Polycaprolactone electrospun fiber scaffold loaded with iPSCs-NSCs and ASCs as a novel tissue engineering scaffold for the treatment of spinal cord injury. Int. J. Nanomed..

[105] Pinar E., Sahin A., Unal S., Gunduz O., Harman F., Kaptanoglu E. (2022). The effect of polycaprolactone/graphene oxide electrospun scaffolds on the neurogenic behavior of adipose stem cells. Eur. Polym. J..

[106] Zhang S., Wang X.-J., Li W.-S., Xu X.-L., Hu J.-B., Kang X.-Q., Qi J., Ying X.-Y., You J., Du Y.-Z. (2018). Polycaprolactone/polysialic acid hybrid, multifunctional nanofiber scaffolds for treatment of spinal cord injury. Acta Biomater..

[107] Ma Y.-H., Shi H.-J., Wei Q.-S., Deng Q.-W., Sun J.-H., Liu Z., Lai B.-Q., Li G., Ding Y., Niu W.-T. (2021). Developing a mechanically matched decellularized spinal cord scaffold for the in situ matrix-based neural repair of spinal cord injury. Biomaterials.

[108] Qian Y., Han Q., Zhao X., Song J., Cheng Y., Fang Z., Ouyang Y., Yuan W.-E., Fan C. (2018). 3D melatonin nerve scaffold reduces oxidative stress and inflammation and increases autophagy in peripheral nerve regeneration. J. Pineal Res..

[109] Zhai H., Zhou J., Xu J., Sun X., Xu Y., Qiu X., Zhang C., Wu Z., Long H., Bai Y. (2020). Mechanically strengthened hybrid peptide-polyester hydrogel and potential applications in spinal cord injury repair. Biomed. Mater..

[110] Wang M., Wang C., Chen M., Luo M., Chen Q., Lei B. (2022). Mechanics-electro-adaptive multifunctional bioactive nanocomposites hydrogel for inducing spinal cord regeneration. Chem. Eng. J..

[111] Shen H., Xu B., Yang C., Xue W., You Z., Wu X., Ma D., Shao D., Leong K., Dai J. (2022). A DAMP-scavenging, IL-10-releasing hydrogel promotes neural regeneration and motor function recovery after spinal cord injury. Biomaterials.

[112] Pina S., Ribeiro V.P., Marques C.F., Maia F.R., Silva T.H., Reis R.L., Oliveira J.M. (2019). Scaffolding Strategies for Tissue Engineering and Regenerative Medicine Applications. Materials.

[113] Ebhodaghe S.O. (2021). Natural Polymeric Scaffolds for Tissue Engineering Applications. J. Biomater. Sci. Polym. Ed..

[114] Yeh J.-Z., Wang D.-H., Cherng J.-H., Wang Y.-W., Fan G.-Y., Liou N.-H., Liu J.-C., Chou C.-H. (2020). A Collagen-Based Scaffold for Promoting Neural Plasticity in a Rat Model of Spinal Cord Injury. Polymers.

[115] Yin W., Li X., Zhao Y., Tan J., Wu S., Cao Y., Li J., Zhu H., Liu W., Tang G. (2018). Taxol-modified collagen scaffold implantation promotes functional recovery after long-distance spinal cord complete transection in canines. Biomater. Sci..

[116] Li L., Xiao B., Mu J., Zhang Y., Zhang C., Cao H., Chen R., Patra H.K., Yang B., Feng S. (2019). A MnO2 Nanoparticle-Dotted Hydrogel Promotes Spinal Cord Repair via Regulating Reactive Oxygen Species Microenvironment and Synergizing with Mesenchymal Stem Cells. ACS Nano.

[117] Kourgiantaki A., Tzeranis D.S., Karali K., Georgelou K., Bampoula E., Psilodimitrakopoulos S., Yannas I.V., Stratakis E., Sidiropoulou K., Charalampopoulos I. (2020). Neural stem cell delivery via porous collagen scaffolds promotes neuronal differentiation and locomotion recovery in spinal cord injury. NPJ Regen. Med..

[118] Li X., Fan C., Xiao Z., Zhao Y., Zhang H., Sun J., Zhuang Y., Wu X., Shi J., Chen Y. (2018). A collagen microchannel scaffold carrying paclitaxel-liposomes induces neuronal differentiation of neural stem cells through Wnt/β-catenin signaling for spinal cord injury repair. Biomaterials.

[119] You K., Chang H., Zhang F., Shen Y., Zhang Y., Cai F., Liu L., Liu X. (2019). Cell-seeded porous silk fibroin scaffolds promotes axonal regeneration and myelination in spinal cord injury rats. Biochem. Biophys. Res. Commun..

[120] Lai B.-Q., Bai Y.-R., Han W.-T., Zhang B., Liu S., Sun J.-H., Liu J.-L., Li G., Zeng X., Ding Y. (2022). Construction of a niche-specific spinal white matter-like tissue to promote directional axon regeneration and myelination for rat spinal cord injury repair. Bioact. Mater..

[121] Lemos N., Fernandes G.L., Ribeiro A.M., Maia-Lemos P.S., Contiero W., Croos-Bezerra V., Tomlison G., Faber J., Oliveira A.S.B., Girão M.J.B.C. (2022). Rehabilitation of People With Chronic Spinal Cord Injury Using a Laparoscopically Implanted Neurostimulator: Impact on Mobility and Urinary, Anorectal, and Sexual Functions. Neuromodul. Technol. Neural Interface.

[122] Adeel M., Lai C.-H., Lin B.-S., Chan W.P., Liou J.-C., Wu C.-W., Peng C.-W. (2022). Effects of paired stimulation with specific waveforms on cortical and spinal plasticity in subjects with a chronic spinal cord injury. J. Formos. Med. Assoc..

[123] Olmsted Z.T., Stigliano C., Scimemi A., Wolfe T., Cibelli J., Horner P.J., Paluh J.L. (2021). Transplantable human motor networks as a neuron-directed strategy for spinal cord injury. iScience.

[124] Kubelick K.P., Emelianov S.Y. (2020). Prussian blue nanocubes as a multimodal contrast agent for image-guided stem cell therapy of the spinal cord. Photoacoustics.

